# Comparative Assessment of Transmission-Blocking Vaccine Candidates against *Plasmodium falciparum*

**DOI:** 10.1038/srep11193

**Published:** 2015-06-11

**Authors:** M. C. Kapulu, D. F. Da, K. Miura, Y Li, A. M. Blagborough, T. S. Churcher, D. Nikolaeva, A. R. Williams, A. L. Goodman, I. Sangare, A. V. Turner, M. G. Cottingham, A. Nicosia, U. Straschil, T. Tsuboi, S. C. Gilbert, Carole A. Long, R. E. Sinden, S. J. Draper, A. V. S. Hill, A. Cohuet, S. Biswas

**Affiliations:** 1 Jenner Institute, University of Oxford, Oxford, OX3 7DQ, UK; 2 Institut de Recherche en Sciences de la Santé (IRSS) et Institut de Recherche pour le Développement (IRD) - UMR MIVEGEC, Bobo-Dioulasso, Burkina Faso; 3 Laboratory of Malaria and Vector Research, National Institute of Allergy and Infectious. Disease / National Institutes of Health, Rockville, Maryland, USA; 4 Division of Cell and Molecular Biology, Imperial College London, London, UK; 5 Department of Infectious Disease Epidemiology, Imperial College London, London, UK; 6 ReiThera Srl (former Okairos Srl), Rome, Italy; 7 CEINGE, Naples, Italy; 8 Department of Molecular Medicine and Medical Biotechnology, University of Naples Federico II, Naples, Italy; 9 Cell-Free Science and Technology Research Centre, Ehime University, Ehime, Japan

## Abstract

Malaria transmission-blocking vaccines (TBVs) target the development of *Plasmodium* parasites within the mosquito, with the aim of preventing malaria transmission from one infected individual to another. Different vaccine platforms, mainly protein-in-adjuvant formulations delivering the leading candidate antigens, have been developed independently and have reported varied transmission-blocking activities (TBA). Here, recombinant chimpanzee adenovirus 63, ChAd63, and modified vaccinia virus Ankara, MVA, expressing AgAPN1, Pfs230-C, Pfs25, and Pfs48/45 were generated. Antibody responses primed individually against all antigens by ChAd63 immunization in BALB/c mice were boosted by the administration of MVA expressing the same antigen. These antibodies exhibited a hierarchy of inhibitory activity against the NF54 laboratory strain of *P. falciparum* in *Anopheles stephensi* mosquitoes using the standard membrane feeding assay (SMFA), with anti-Pfs230-C and anti-Pfs25 antibodies giving complete blockade. The observed rank order of inhibition was replicated against *P. falciparum* African field isolates in *A. gambiae* in direct membrane feeding assays (DMFA). TBA achieved was IgG concentration dependent. This study provides the first head-to-head comparative analysis of leading antigens using two different parasite sources in two different vector species, and can be used to guide selection of TBVs for future clinical development using the viral-vectored delivery platform.

Malaria is still one of the world’s major infectious diseases and exerts a devastating burden on global public health. The development of an effective vaccine against *Plasmodium falciparum* remains an important but elusive goal; to date, only low-level efficacies have been achieved by a handful of approaches in clinical studies^1^. The most advanced malaria vaccine candidate, currently in Phase III clinical trials, is based on the circumsporozoite protein (CSP), and aims to prevent infection of the vaccinated host^2^. Transmission-blocking vaccines (TBVs) target antigens expressed by the transmissible sexual-stages of the parasite, as well as those expressed by the mosquito, and aim to reduce and/or block transmission to the vertebrate host^3^,^4^. Pre-clinical studies investigating TBVs have led to the development of a number of *P. falciparum* antigens as vaccine candidates of which Pfs230, Pfs25, and Pfs48/45 are well characterized. Different vaccine platforms utilizing these antigens have been used to raise antibodies with demonstrable transmission-blocking activity (TBA)^5^. However, to date only Pfs25 and its ortholog in *P. vivax*, Pvs25, have been tested in Phase Ia clinical trials^6^,^7^.

Pfs25 is expressed during macrogametogenesis within the mosquito midgut and persists throughout zygote, ookinete and early oocyst development. Pfs25 antigen-specific IgG demonstrate TBA and antibody titer correlates with ability to block parasite infectivity^8^,^9^,^10^. On the other hand, Pfs230 and Pfs48/45 are expressed on the surface of gametocytes within the human host and have a role in gamete-gamete recognition within the vector blood meal^11^,^12^. Individuals living in malaria endemic areas can naturally develop antibodies against these two antigens^13^,^14^,^15^,^16^.

Recently it has been shown that mosquito midgut ligands that mediate parasite invasion can also be targeted as TBV candidate antigens. Expressed midgut proteins of *Anopheles stephensi and A. gambiae* such as carboxypeptidase B^4^,^17^,^18^ and a 135 amino acid fragment of the *A. gambiae* midgut glycoprotein, alanyl aminopeptidase N1 (AgAPN1) have been developed as potential TBV candidates. Antibodies raised against this fragment of AgAPN1 have demonstrated TBA in studies using both *P. falciparum* and *P. berghei* parasites in two different anopheline species, *A. gambiae* and *A. stephensi*^19^.

Despite the lack of any reported comparison of TBA, Pfs230, Pfs25 and Pfs48/45 are currently considered the leading TBV candidate antigens. Their ability to induce a transmission-blocking response has been examined in different laboratories, and the antigens have been expressed mainly as recombinant proteins produced in a variety of heterologous expression systems as well as delivered using vectored vaccine technologies^20^. The development of some TBVs, especially members of the six-cysteine family of proteins Pfs230 and Pfs48/45, has been significantly hampered by the difficulties associated with the expression of full-length, correctly folded native protein. The induction of transmission-blocking antibodies targeting these antigens is critically dependent on conformationally correct immunogens^21^,^22^. In the case of Pfs230, the region spanning amino acids 443-1132, referred to as region C (Pfs230-C), has been shown to induce the most potent TBA^23^. For Pfs48/45, codon harmonization of the native sequence has led to the expression of full-length protein, that induces antibodies giving greater than 95% inhibition of oocyst formation in both rodents and primates when formulated in the Montanide ISA-51 adjuvant^24^. Pfs25 protein has been used alone or conjugated to highly immunogenic carriers such as outer-membrane protein complex (OMPC) of *Neisseria meningitidis* serogroup B^25^. Pfs25 has also been delivered in a heterologous prime-boost regime using human adenovirus serotype 5 (HuAd5) and modified vaccinia virus Ankara (MVA) viral-vectors eliciting antibodies that exhibit TBA^26^.

Traditionally, viral vectors have been used in prime-boost regimes primarily for the induction of antigen-specific T cells, but HuAd5 and ChAd63, in a prime-boost regime with MVA, have also been used to induce functional antibodies against blood-stage malaria antigens in both pre-clinical and Phase I/IIa clinical studies^27^,^28^,^29^,^30^. Although potent as an antigen delivery vector, HuAd5 has not been favored for translation into the clinic due to the presence of neutralizing antibodies from pre-existing infections in individuals living in malaria endemic areas^31^. To circumvent this, chimpanzee adenoviruses (which are reported to have lower pre-existing neutralizing antibodies) have been used^31^. This human-compatible delivery system provides a robust and versatile platform to enable the testing and screening of TBV candidate antigens expressed *in situ*, while avoiding potential differences in potency caused by variable purification of expressed proteins.

In this study we have used the ChAd63-MVA heterologous prime-boost regime to compare four TBV candidate antigens head-to-head to identify antigen(s) for future clinical development in this viral vectored vaccination platform. We investigated AgAPN1, Pfs25, Pfs48/45, and region C of Pfs230 (Pfs230-C), and we assessed the ability of vaccine-induced IgG against these antigens to inhibit the development of *P. falciparum* oocysts within the mosquito, in order to guide prioritization of these candidates for clinical development in this delivery platform. We report that vaccine-induced IgG against Pfs230-C and Pfs25 completely blocked transmission of *P. falciparum* both the laboratory clone NF54 and field isolates from Burkina Faso.

## Results:

### Generation and expression of TBV candidate antigens in ChAd63 and MVA viral vectors

Recombinant ChAd63 and MVA vectors expressing Pfs25 were generated previously^26^. We designed and generated recombinant ChAd63 and MVA vaccines expressing three additional TBV candidate antigens: AgAPN1 based on the genome sequence of *A. gambiae* PEST strain; Pfs230-C and two versions of Pfs48/45 (Pfs48/45_−NGln_ and Pfs48/45_+NGln_) based on the genomic sequence of the *P. falciparum* 3D7 clone (Table 1). For AgAPN1 and Pfs48/45 the signal peptide and glycosylphosphatidylinositol (GPI) anchor were not included in the construct. The inserts were used to generate recombinant ChAd63 and MVA expressing the individual antigens (Supplementary Information).

**Figure 1:**
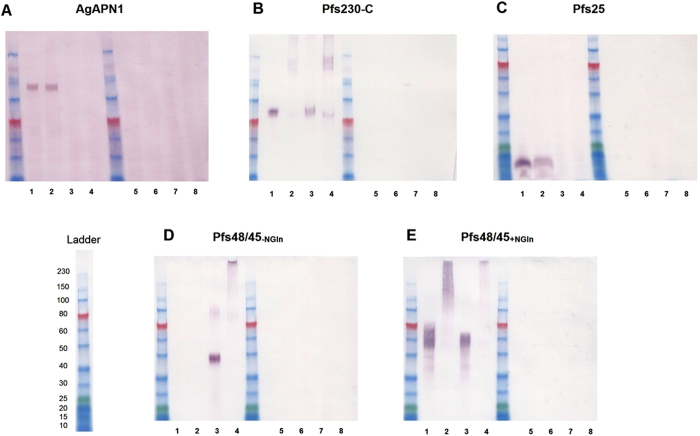
Expression of TBV candidate antigens in adherent HEK293 cells. pENTR4-LPTOS shuttle plasmid DNA expressing (A) AgAPN1, (B) Pfs230-C, (C) Pfs25, (D) Pfs48/45−NGln, and (E) Pfs48/45+NGln was used to transfect HEK293 cells and the supernatant and cell lysate were harvested. 5 μg protein was loaded per lane and after staining the western blots were developed for 5 min. The figure shows western blots using day70 anti-serum (pooled from 5 mice) collected after ChAd63-MVA vaccination against the respective antigens. Untransfected supernatant and cell lysate were used as negative controls. Lanes: 1- antigen-specific supernatant in reducing buffer; 2- antigen-specific supernatant in non-reducing buffer; 3- antigen-specific lysate in reducing buffer; 4- antigen-specific lysate in non-reducing buffer; 5- untransfected supernatant in non-reducing buffer; 6- untransfected supernatant in reducing buffer; 7- untransfected lysate in non-reducing buffer; 8- untransfected lysate in reducing buffer. A protein ladder is included in the figure for reference.

To test the expression of the antigens, pENTR4-LPTOS shuttle plasmid DNA expressing each individual antigen (under the control of the human cytomegalovirus (CMV) promoter^32^) was used to transfect HEK293 cells. Immunoblots of supernatants and cell lysates showed expression of the antigens at the expected size (AgAPN1 at 112 kDa, Pfs230-C at 83.5 kDa, Pfs25 at 18.8 kDa and Pfs48/45_−NGln_ at 46 kDa)^26^ (Fig. 1). For Pfs48/45_+NGln_ the molecular weight of the expressed protein was higher than expected (~60 kDa) possibly due to glycosylation. In Supplementary Fig. 1D when the supernatant was treated with Peptide-N-Glycosidase (optimized for the efficient release of N-linked oligosaccharides) the protein was expressed at the predicted size (46 kDa). Recombinant proteins were detected in supernatant for AgAPN1, Pfs230-C, Pfs25 and Pfs48/45_+NGln_ suggesting that the incorporation of the tPA signal peptide leads to secretion of the antigen which is thought to be optimal for antibody induction^33^. Higher molecular weight bands were observed for Pfs230-C and Pfs48/45_+NGln_ which could be dimers or a result of protein aggregation.

### Heterologous prime-boost immunization induces IgG responses against TBV candidate antigens

We have previously shown that anti-Pfs25 antibodies, induced after vaccination with the HuAd5-MVA heterologous prime-boost vaccine regime, are functional and block the transmission of *P. falciparum* in a standard membrane feeding assay (SMFA)^26^ A pilot study was performed to test whether the ChAd63-MVA heterologous prime-boost regime was also able to induce antibodies to AgAPN1, Pfs230-C, Pfs48/45_−NGln_, and Pfs48/45_+NGln_. Antigen-specific IgG was induced after the priming immunization with ChAd63 expressing the individual antigens, which was boosted significantly following the administration of the corresponding recombinant MVA (data not shown). Immune responses observed in the pilot study justified further testing of the longevity of vaccine-induced antibody responses. It has been previously shown that responses to Pfs25 eight weeks post-prime are similar whether primed with HuAd5 or ChAd63^26^.

**Figure 2:**
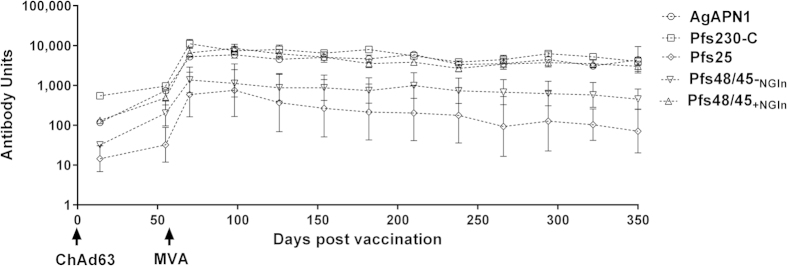
Antigen-specific IgG responses in mice after immunization with ChAd63-MVA expressing TBV antigens. BALB/c mice (*n* = 5/group) were primed with 1 × 10^8^ infectious units (IFU) of ChAd63 (day 0) and boosted with 1 × 10^7^ plaque forming units (PFU) of MVA (day 56) expressing the individual antigens AgAPN1, Pfs230-C, Pfs25, Pfs48/45_−NGln_, and Pfs48/45_+NGln_. The figure shows the total IgG response measured by standardized ELISA on days 14, 55, 70 and thereafter every 28 days up to day 350. IgG responses were log-transformed and expressed as antibody units (AU) represented as mean ± SEM. The AU are not directly comparable between antigens, except for Pfs48/45 where the same coating antigen and ELISA method was used. Wilcoxon matched-pairs signed rank test was used to assess differences between day 55 and day 70 responses, whilst Friedman test with Dunn’s multiple comparison test was used to determine significance between day 70 and day 350. *p* ≤ 0.05 was considered significant (see Results text).

IgG responses in this study were assessed at days 14 and 55 post-prime and day 14 post-boost (Fig. 2). Using Wilcoxon matched-pairs signed rank test the corresponding recombinant MVA significantly boosted the response to AgAPN1 (*p* = 0.008), Pfs230-C (*p* = 0.03) and Pfs48/45_+NGln_ (*p* *=* 0.03). The increase in Pfs25 antibody response after the MVA boost was not statistically significant (*p* = 0.06). We have previously reported that MVA significantly boosts the antibody response to Pfs25 primed by HuAd5^26^ and this is reproducible in other experiments when primed with ChAd63. In this particular experiment there was variability in the antibody response in the group of animals vaccinated against Pfs25 but we have repeated this experiment and seen less variability. The Pfs48/45_−NGln_ (*p* = 0.09) antibody response was not significantly boosted by the MVA. When tested in the ELISA against recombinant protein produced using the wheat germ cell-free protein expression system, there was a consistent trend that the vaccine-induced IgG response against Pfs48/45_+NGln_ compared to Pfs48/45_−NGln_ (day 70) was higher. In order to assess the longevity of the antibody responses, the animals were monitored for ten and a half months post-boost. The responses were well maintained (no significant difference between day 70 and day 350 by Friedman test with Dunn’s multiple comparison test), for all the antigens except Pfs25.

### *Vaccine-induced antibody responses recognize native parasite and mosquito proteins*

**Figure 3:**
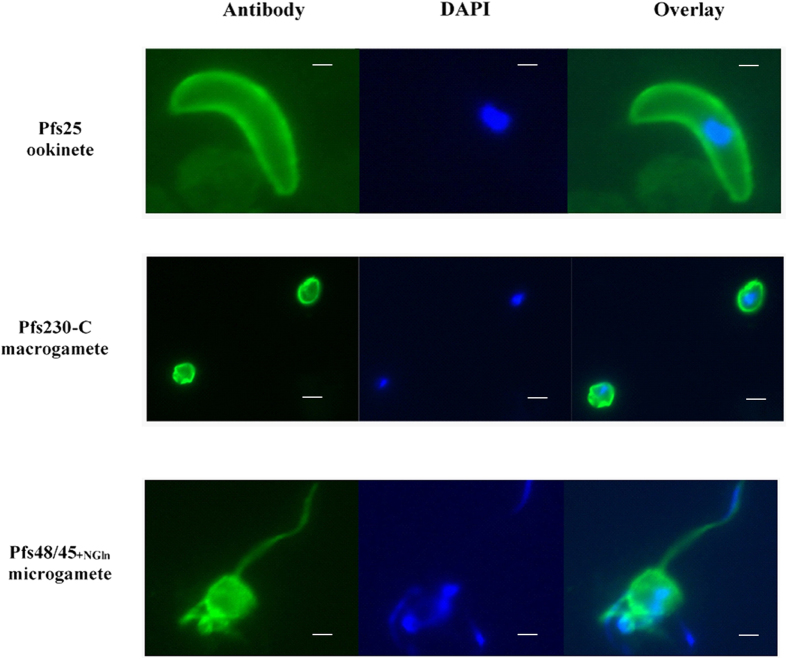
Reactivity of vaccine-induced antibodies against Pfs230-C, Pfs25 and Pfs48/45+_NGln_ to native parasite antigen. *In vitro* cultured Pfs25DR3 transgenic *P. berghei* ookinetes or *P. falciparum* NF54 microgametes and macrogametes were stained with purified total IgG from pooled serum (*n* = 5) against Pfs25 (top panel) or antisera against Pfs230-C (middle panel) and Pfs48/45_+NGln_ (bottom panel). Antibody binding was detected by Alexa Fluor® 488-conjugated goat anti-mouse IgG (green) and the DNA was stained with DAPI (blue). Scale bars, 2 μm.

To determine whether antibodies induced against Pfs230-C, Pfs25, Pfs48/45_−NGln_, and Pfs48/45_+NGln_ recognize the respective native parasite proteins, indirect immunofluorescence assays (IFA) were performed on parasite preparations (mature *P. falciparum* gametocytes or Pfs25DR3 *P. berghei* ookinetes) (Fig. 3). Surface staining of macrogametes with vaccine induced anti-Pfs230-C antibodies and ookinetes with anti-Pfs25 antibodies confirmed the ability of the antibodies to recognize native parasite protein. The anti-Pfs48/45_+NGln_ antibodies stained the surface of exflagellating male gametes but no staining was observed with Pfs48/45_−NGln_ antiserum (data not shown). Naïve and viral vector control serum (mice immunized with ChAd63-MVA expressing GFP) were used as negative controls and showed undetectable levels of staining (data not shown).

**Figure 4:**
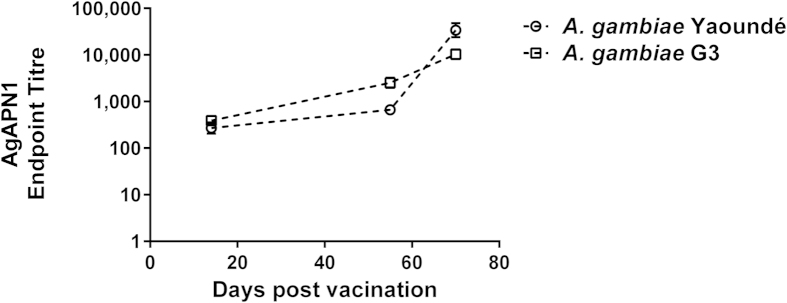
Reactivity of vaccine-induced antibodies to *A. gambiae* midgut lysate. BALB/c mice (*n* = 5) were vaccinated with ChAd63 expressing AgAPN1 (day 0) and boosted with the respective MVA (day 56). Antibody responses were measured on days 14, 55 and 70 by endpoint ELISA using midguts lysates obtained from *A. gambiae* Yaoundé and G3 strains. The data were log-transformed and are represented as mean ± SEM for the different time-points.

The anti-AgAPN1 serum recognized native antigen in an ELISA against midgut lysate from two strains of *A. gambiae,* Yaoundé and G3 (Fig. 4). Anti-AgAPN1 serum also recognized native AgAPN1 by western blot using midgut lysate from both *A. gambiae* Yaoundé (reducing and non-reducing conditions) and *A. stephensi* SDA500 (reducing condition) with similar banding patterns previously observed and described^19^ (Supplementary Fig. 1A). We also tested the anti-Pfs48/45_−NGln_ and anti-Pfs48/45_+NGln_ serum in a western blot using *P. falciparum* gametocyte lysate and they recognized a protein of 46 kDa (Supplementary Fig. 1B and C respectively).

### Inhibition of P. falciparum NF54 development in A. stephensi by vaccine-induced IgG

Vaccine-induced antibodies against Pfs230-C, Pfs25, and Pfs48/45 have shown varying TBA against *P. falciparum* depending on the antigen delivery system used^24^,^34^. Antibodies against AgAPN1 have been shown to inhibit the infectivity of both *P. berghei* and *P. falciparum*^19^,^35^. Here we performed a head-to-head comparison of the ability of antigen-specific IgG (induced against each antigen with a common vaccine platform) to reduce the intensity and prevalence of *P. falciparum* NF54 parasite infection in a laboratory-reared vector (*A. stephensi*) using SFMA. TBA of antibodies against Pfs230-C has been shown previously to be complement-dependent^36^,^37^, and thus all assays were performed in the presence of complement. To be able to distinguish antibody-specific inhibition from non-specific effects of mouse serum, total purified IgG was used.

**Figure 5:**
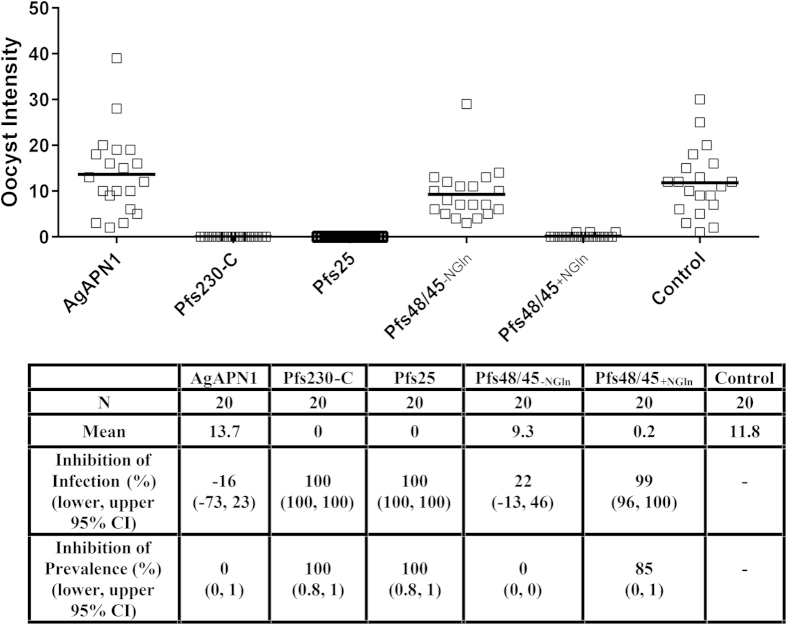
Effect of mouse IgG induced by ChAd63-MVA immunization on P. *falciparum* NF54 parasite infectivity in *A. stephensi* mosquitoes. Pooled day 70 serum (*n* = 5) was used to purify total IgG against each of the antigens. 655 μg/mL of each of the total IgG was mixed with *P. falciparum* NF54 cultured gametocytes and fed to *A. stephensi* mosquitoes (*n* = 20) in SMFA. Midguts were dissected 7 days post-feeding. Data points represent the number of oocysts in individual mosquitoes and the lines show the arithmetic mean (from one experiment). The table shows the number of mosquitoes dissected (N) per antigen, mean number of oocysts, percent inhibition of infection intensity and prevalence calculated relative to the IgG from mice immunized with vectors expressing GFP. GLMM with a zero-inflated negative binomial error structure was used to describe oocyst intensity whilst a binomial error structure was used to determine oocyst presence or absence. Uncertainty was generated using bootstrapping methodology. *p* ≤ 0.05 was considered significant.

IgG against Pfs230-C and Pfs25 showed complete inhibition of oocyst intensity and prevalence (*p* < 0.00001 for both) whereas anti-Pfs48/45_+NGln_ IgG showed 99% inhibition of oocyst intensity and 85% inhibition of oocyst prevalence (*p* < 0.00001 for both) (Fig. 5). The IgG were tested in another independent SMFA and anti-Pfs230-C, Pfs25 and Pfs48/45_+NGln_ all gave >89% of oocyst intensity (Supplementary Fig. 2). There was no statistically significant inhibition seen with anti-AgAPN1 (tested in two independent feeds) and anti-Pfs48/45_−NGln_ IgG at the same IgG concentration.

### Inhibition of African field isolates of P. falciparum in A. gambiae by vaccine-induced IgG

We also tested the IgG in a direct membrane feeding assay (DMFA) using *P. falciparum* parasites collected from gametocyte donors (children aged between 5 and 11 years) in Bobo-Dioulasso, Burkina Faso. This was tested using laboratory-adapted *A. gambiae s.s*, M molecular form, one of the predominant vectors for malaria transmission in this region. Although most TBV antigens have limited polymorphisms^38^, we wanted to assess whether the efficacy and the ranking of the antigens against *P. falciparum* NF54 would be replicated against field isolates in a second vector species.

**Figure 6:**
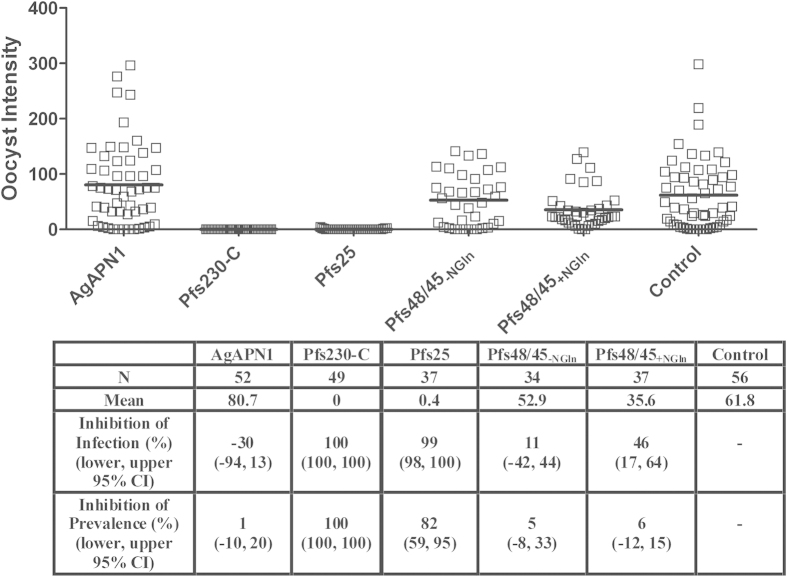
Effect of mouse IgG induced by ChAd63-MVA immunization on infectivity of *P. falciparum* field isolate to A. *gambiae* mosquitoes. The purified IgG (from pooled serum (*n* = 5)) for each of the antigens and gametocyte positive donor (BF001) RBCs were used in a DMFA. The gametocytaemia of the donor was 30 gametocytes per 1000 leukocytes and the ratio of male:female gametocytes was 1.44. The plasma from the gametocyte positive donor blood was replaced with 500 μg/mL IgG and immediately fed to *A. gambiae* mosquitoes. Midguts were dissected 7 days post-feeding. Data points represent the number of oocysts in individual mosquitoes and the lines show the arithmetic mean. The table shows the number of mosquitoes dissected (N) per antigen, mean number of oocysts, percent inhibition of infection intensity and prevalence calculated relative to the IgG from mice immunized with vectors expressing GFP. GLMM with a zero-inflated negative binomial error structure was used to describe oocyst intensity whilst a binomial error structure was used to determine oocyst presence or absence. Uncertainty was generated using bootstrapping methodology. *p* ≤ 0.05 was considered significant.

Significant inhibition of oocyst intensity and prevalence consistent with the previous assay (Fig. 5) was seen with anti-Pfs230-C (complete inhibition, *p* < 0.00001 for both) and anti-Pfs25 (99%, and 81.8% respectively, *p* < 0.00001 for both) IgG (Fig. 6). Anti-Pfs48/45_+NGln_ IgG only significantly inhibited the oocyst intensity (45.5%, *p* = 0.007) and not prevalence in this experiment (Fig. 6). There was no significant inhibition seen with anti-AgAPN1 and anti-Pfs48/45_−NGln_ IgG. The percent inhibition seen with anti-Pfs48/45_+NGln_ IgG was lower than anti-Pfs230-C and anti-Pfs25 IgG.

**Figure 7:**
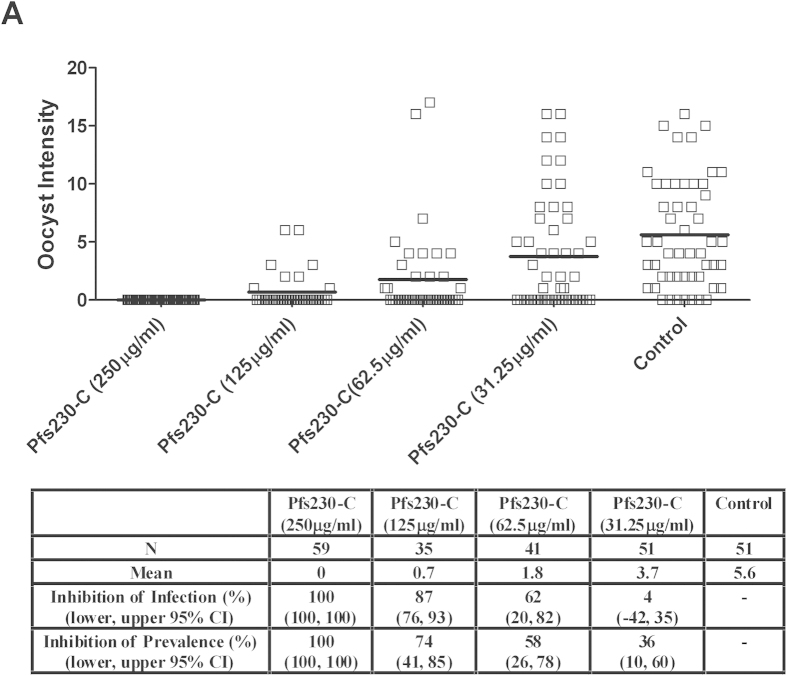
TBA of anti-Pfs230-C at different concentrations in DMFA. Membrane feeds were performed as described in Fig. 6 using a range of anti-Pfs230-C (7) and anti-Pfs25 (8) IgG concentrations (shown in brackets on the x-axis). The gametocytaemia of the donor (BF002) was 10 gametocytes per 1000 leukocytes and the ratio of male:female gametocytes was 0.59. The IgG was tested with non-heat-inactivated AB+ serum as a source of complement. Data points represent the number of oocysts in individual mosquitoes and the lines show the arithmetic mean. The table shows the number of mosquitoes dissected (N) per antigen, mean number of oocysts, percent inhibition of infection intensity and prevalence calculated relative to the IgG from mice immunized with vectors expressing GFP. GLMM with a zero-inflated negative binomial error structure was used to describe oocyst intensity whilst a binomial error structure was used to determine oocyst presence or absence. Uncertainty was generated using bootstrapping methodology. *p* ≤ 0.05 was considered significant.

**Figure 8:**
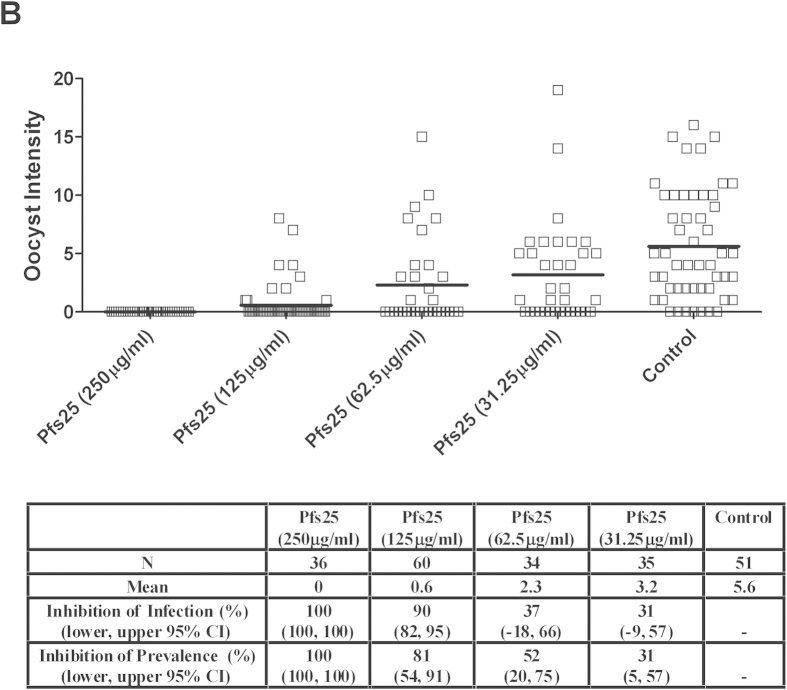
TBA of anti-Pfs25 IgG at different concentrations in DMFA. Membrane feeds were performed as described in Fig. 6 using a range of anti-Pfs230-C (7) and anti-Pfs25 (8) IgG concentrations (shown in brackets on the x-axis). The gametocytaemia of the donor (BF002) was 10 gametocytes per 1000 leukocytes and the ratio of male:female gametocytes was 0.59. The IgG was tested with non-heat-inactivated AB+ serum as a source of complement. Data points represent the number of oocysts in individual mosquitoes and the lines show the arithmetic mean. The table shows the number of mosquitoes dissected (N) per antigen, mean number of oocysts, percent inhibition of infection intensity and prevalence calculated relative to the IgG from mice immunized with vectors expressing GFP. GLMM with a zero-inflated negative binomial error structure was used to describe oocyst intensity whilst a binomial error structure was used to determine oocyst presence or absence. Uncertainty was generated using bootstrapping methodology. *p* ≤ 0.05 was considered significant.

Anti-Pfs230-C and anti-Pfs25 IgG showed superior TBA (>99% inhibition of oocyst intensity) than the other antigens (Fig. 6). We thus investigated the effect of reducing the concentration of anti-Pfs230-C and anti-Pfs25 IgG in the membrane feeding assay (Figs 7 and 8). When tested at 250 μg/mL against a different gametocyte donor (BF002), both anti-Pfs230-C and anti-Pfs25 IgG still gave complete blockade. Significant inhibition of oocyst intensity and prevalence was also seen in this experiment with anti-Pfs230-C (*p* = 0.007 and *p* < 0.00001 respectively) and anti-Pfs25 (*p* < 0.00001 for both) IgG, with the same results observed at both 62.5 μg/mL and 125 μg/mL concentrations.

The anti-AgAPN1, anti-Pfs48/45_−NGln_ and anti-Pfs48/45_+NGln_ IgG were also tested in two further experiments using gametocytes from two different donors (BF003 and BF004). In this study anti-AgAPN1 IgG still showed no significant effect on oocyst prevalence in any of the experiments. However, anti-AgAPN1 IgG gave significant inhibition of oocyst intensity in one experiment (donor BF004, Control 2 in Supplementary Fig. 3) but this result was not replicated in any other feeds performed. The anti-Pfs48/45_−NGln_ (82% and 78%, *p* = 0.01 and *p* < 0.0001 respectively) and anti-Pfs48/45_+NGln_ (100%, *p* < 0.0001 for both) IgG showed significant inhibition in one assay (Control 2, Supplementary Fig. 3), whilst only anti-Pfs48/45_−NGln_ IgG showed significant inhibition of oocyst intensity (55%, *p* *=* 0.02) in the other (Control 1, Supplementary Fig. 3). Control 2 had a lower oocyst number (8.5 oocysts) which could be due to the difference in the gametocytes, either amount ingested or sex ratio. The difference in inhibition could also be due to polymorphism in Pfs48/45, which has been reported previously^38^,^39^ or the number of parasite clones (multiplicity of infection), which might be an additional source of Pfs48/45 polymorphism in a single feed.

In light of the above results, we also expressed two smaller fragments of AgAPN1 using recombinant viral vectors; one corresponding to the N terminal fragment (amino acids 61-195) reported by Dinglasan *et al.*^19^ (AgAPN1 Nterm) and the other consisting of amino acids 20-303 based on the domain structure of AgAPN1 (AgAPN1 DomI). Total IgG was purified from sera (d70) from mice vaccinated with ChAd63 and MVA expressing AgAPN1, AgAPN1 DomI and AgAPN1 Nterm and tested in a SMFA at 750 μg/mL and 375 μg/mL (Supplementary Fig. 4). Anti-AgAPN1 and anti-AgAPN1 DomI IgG showed no significant effect on oocyst intensity and oocyst prevalence. Anti-AgAPN1 Nterm IgG gave significant inhibition of oocyst intensity (48%, *p* = 0.02) at 375 μg/mL but not 750 μg/mL, suggesting marginal TBA.

## Discussion:

Here we describe a head-to-head comparison of the leading transmission-blocking vaccine candidate antigens using a human-compatible vaccine platform (heterologous prime-boost viral vectored delivery). TBV candidate antigens have been previously assessed individually for their ability to block parasite infectivity to mosquitoes using a wealth of different delivery platforms. In this comparative study, anti-Pfs230-C and anti-Pfs25 antibodies induced after vaccination with this viral vectored regime showed superior transmission-blocking activity compared to Pfs48/45 and AgAPN1 (each antigen construct (with the exception of Pfs48/45_−Ngln_) was tested a total of 2–4 times by SMFA and 3 times by DMFA with very similar results). IgG raised against Pfs230-C and Pfs25 have consistently shown better (>99%) TBA on both parasite infectivity and prevalence using both laboratory and field isolates of *P. falciparum* in *A. stephensi* and *A. gambiae* respectively. We have previously reported the use of this delivery system to induce functional antibodies to Pfs25 when expressed from a human adenovirus (HuAd5) and MVA^26^. This work provides additional evidence that the viral vectored delivery platform can induce functional antibodies against a range of candidate TBV antigens. This platform has been used successfully to induce functional antibodies against leading blood-stage malaria antigens in humans^29^,^30^,^40^, and used successfully to identify novel target antigens for further development^41^.

The development of certain TBV candidate antigens has been hampered by the inability to express full-length correctly folded proteins, like the sexual-stage cysteine-rich protein Pfs48/45 which has conformation-dependent target epitopes^42^,^43^. The viral vector delivery platform enabled us to express these antigens and induce antigen-specific antibodies. The ChAd63 vaccine primed a detectable antibody response that increased over time and was boosted after the administration of MVA expressing the respective antigen. These data are in accordance with previous findings where an 8 week interval between prime and boost was shown to induce high-titer antibodies against blood-stage malaria antigens and Pfs25^26^,^28^.

The antibody response is sustained for all the antigens (except Pfs25) using this delivery system, without the need for additional booster vaccinations after the initial prime-boost regime in mice. A sustained antibody response for at least one transmission season is likely to be essential to enable effective blockade in endemic areas. The decline in the antibody response to Pfs25 compared to the other antigens could be in part due to the inherent properties of the antigen. It has been reported previously that antibodies against Pfs25 decline to 50–70% of the peak response in mice after six months of follow up^25^. To address this, Pfs25 protein has been conjugated to several carrier proteins in an attempt to make it more immunogenic. A sustained antibody response was achieved in rhesus monkeys only after chemically conjugating Pfs25 to the OMPC of *N. meningitidis* serogroup B and adsorption to aluminum hydroxyphosphate^25^. A recent study has demonstrated that the induction of CD4^+^ T cells following an adjuvanted influenza vaccine predicted the persistence of the antibody response^44^, and several pre-clinical and clinical studies have shown that viral vectors induce strong T cell responses^29^,^45^. We did not assess the IgG subclass responses in this study but we have published previously that this viral vectored regime induces a mixed Th1/Th2 response^26^,^45^. The role of memory B cells which are important for a rapid recall response was also not assessed in this study, but viral vectored vaccination regimes have been shown to induce memory B cells in rhesus macaques^27^ and humans^46^. It will be important to further assess the role of CD4^+^ T cell help and memory B cells in the context of antibody responses to TBV candidate antigens, especially Pfs25 where antibody maintenance may be sub-optimal. In contrast to Pfs25, antibody responses to antigens such as Pfs230 and Pfs48/45 could potentially be boosted by natural infection and gametocyte exposure, but this requires experimental assessment. It has been shown in the *P. berghei* model that antibody responses induced by immunization with DNA encoding Pbs48/45 were boosted after a blood-stage challenge in mice^47^.

Our results suggest that there is a possible effect of N-glycosylation site substitution on conformation, immunogenicity and transmission-blocking activity of Pfs48/45. In previously published studies, expression of Pfs48/45 in vaccinia virus resulted in a glycosylated product that was considerably less immunogenic in mice resulting in a lowered ability to inhibit parasite infectivity to *A. stephensi*^48^,^49^. In contrast to the published data, the IgG against Pfs48/45_−NGln_ (with N-glycosylation site substitutions) did not show any transmission blocking effect in the SMFA against *P. falciparum* NF54, but did recognize antigen in a Western blot. This could be due to the lower antibody response seen with this construct, or due to unintended alterations in functionally important, conformational B cell epitopes caused by amino acid substitutions. In only one of the DMFA did IgG against Pfs48/45_−NGln_ show measurable TBA, but this was marginal and needs further investigation. Polymorphisms in Pfs48/45 have been reported in field isolates and this could explain the variability in TBA seen in the Burkina Faso studies^38^. The variability in TBA of antibodies against Pfs48/45 could also be dependent on the amount and sex ratio of gametocytes ingested. We conclude that further studies need to be done to understand the variability of TBA seen with IgG against Pfs48/45 and could include comparative assessment of the 10C region of Pfs48/45^50^. We are currently investigating further the impact of oocyst numbers in the control mosquitoes and antigen polymorphism on TBA in Burkina Faso. It is critical that such an approach be adopted for the screening of potential TBV candidate antigens as we have recently shown that vaccine efficacy needs to be tested against a range of parasite exposures to interpret laboratory results accurately^51^.

It was surprising that anti-AgAPN1 IgG induced by viral vectored prime-boost vaccination exhibited variable TBA. It has been shown previously that antibodies to an N-terminal fragment of AgAPN1 can block the development of both *P. berghei* and *P. falciparum*^19^,^35^. This difference in TBA could be due to the difference in construct design, although we have shown that the antibodies induced here recognize native antigen in both immunoblots and ELISA which provides evidence for recognition of the target antigen. It could also be due to presence or absence of O-linked glycosylation or due to the masking of transmission-blocking epitopes by other immunodominant epitopes^52^. This antigen remains an attractive candidate due to its effects on diverse *Plasmodium* species in different anopheline species but requires further investigation in this system.

It has been reported that antibodies against Pfs25 and Pfs28 have synergistic TBA when IgG against the two antigens are combined in an SMFA^53^ or when expressed as fusion protein in *Saccharomyces cerevisiae*^54^. Investigation of whether the same synergy could be achieved with anti-Pfs25 and Pfs230-C antibodies will be important, and also ways to combine the two antigens in a single delivery platform. It will also be important in the future to determine the IC_50_ (concentration of antigen-specific antibody required to inhibit 50 percent of oocyst development)^55^ of the antibodies against Pfs230-C and Pfs25 against field isolates, in order to determine the concentration of IgG required for effective blockade in different transmission settings. The viral vector platform used in this study has a proven safety record in clinical trials targeting a range of pathogens^30^,^56^,^57^. The ability to induce antibody responses with functional activity against TBV candidate antigens with inhibitory effects of up to 100% observed for two antigens is very encouraging for further development of these candidate vaccines for clinical trials.

## Methods:

### Design and generation of recombinant viral vectored vaccines

Antigen sequences for AgAPN1, (amino acids (aa) 20–998; AGAP004809, PEST strain), Pfs230 (PF3D7_0209000), Pfs48/45 (aa 28–427; PF3D7_1346700) and Pfs25 (aa 22–192; PF3D7_1031000) were obtained from NCBI protein database. For Pfs230, only region C (aa 443–1132, Pfs230-C) against which antibodies have been shown previously to inhibit parasite development was used^58^,^59^. All putative N-glycosylation sites were changed from Asn-Xaa-Ser/Thr to Gln-Xaa-Ser/Thr. For Pfs48/45 two different constructs were designed; one with all seven N-glycosylation sites^43^ substituted (Pfs48/45_−NGln_) and the other with the native amino acid sequence (Pfs48/45_+NGln_). In addition, two fragments of AgAPN1 were designed corresponding to amino acids 61–195 (AgAPN1 Nterm) and amino acids 20–303 (AgAPN1 DomI). The antigen sequences were codon optimized for expression in humans (GeneArt® Life Technologies, Germany) and the predicted native signal peptide for each antigen was replaced with the human tissue plasminogen activator (tPA) signal peptide^28^. The recombinant viral vaccines were generated as described in Supplementary Information.

### Animal studies and vaccinations

Age-matched female BALB/c mice (Harlan, UK), housed in specific-pathogen free environments, were vaccinated via the intramuscular (i.m.) route using a heterologous prime-boost regime. To determine whether AgAPN1, Pfs230-C, Pfs48/45_−NGln_, and Pfs48/45_+NGln_ were immunogenic when administered in this delivery system, which has been previously shown to elicit anti-Pfs25 specific antibodies^26^, a pilot study was undertaken. In all experiments, animals were vaccinated with a ChAd63 (day 0) priming dose of 1 × 10^8^ IFU and boosted with MVA (day 56) at 1 × 10^7^ PFU expressing the individual test antigens. Control vaccinations were performed with ChAd63 and MVA expressing green fluorescent protein (GFP). Vaccines were prepared in sterile endotoxin-free PBS (Invitrogen, UK). Vaccine-induced antibody responses were assessed on days 14, 55 and 70 and then every month for ten and a half months. All animal experiments and procedures were performed according to the UK Animals (Scientific Procedures) Act Project Licence (PPL 30/2414 and 30/2889) and approved by the Oxford University Local Ethical Review Committee.

### Western Blot Analysis

To determine the expression of the recombinant antigens expressed by the constructs in mammalian cells, 1 × 10^7^ cells/mL HEK293 cells were seeded onto 6 well plates and transfected with pENTR™ (Invitrogen, UK) entry plasmid (containing the tetracycline operator in proximity to the TATA box, pENTR™4LPTOS)^41^) using Lipofectamine™ 2000 (Invitrogen, UK). Cells were incubated overnight at 37 °C and 5% CO_2_. Supernatant and cell lysate were harvested in non-reducing, and reducing, SDS-PAGE loading buffers and boiled at 95 °C for 5 min before running on 8 or 12% polyacrylamide gels (Thermo Scientific, UK) (5 μg/lane). Proteins were transferred onto 0.45 μm nitrocellulose membranes, which were then blocked in 3% BSA/PBS overnight, washed twice with PBS/0.05% Tween^®^20 (PBS/T) and incubated with sera (pooled d70 serum from all mice (*n* = 5) immunized with the respective antigens) diluted 1:100 in 3% BSA/PBS for 1 h. The blots were then washed and incubated for 1 h with donkey anti-mouse IgG AP-conjugated antibody (Jackson ImmunoResearch Laboratories, Inc.) diluted at 1:5000, in 3% BSA/PBS. After repeating the washing procedure, the protein bands were detected using BCIP/NBT alkaline phosphatase substrate (Sigma, UK) and after 5 min the images were scanned using Canon Canoscan LiDe200. Color plus Pre-stained Protein ladders (NEB, UK) were used to estimate the relative protein mobility.

### ELISAs

To detect antigen-specific IgG antibodies induced by vaccination, ELISAs were performed as previously described^10^,^60^. The recombinant proteins for AgAPN1, Pfs230-C and Pfs48/45 were produced using the wheat-germ cell free system^61^ and the Pfs25 protein antigen was provided by Dr Yimin Wu (NIH, USA). A standard reference serum was created using pooled day 70 vaccinated mouse serum with high titers to the respective antigens. The standard reference was used on a standardized ELISA to determine the ELISA units, expressed as antibody units (AU), for each individual antigen. For Pfs25 ELISAs, a previously reported standard serum was used^10^,^25^. A negative control (pooled serum from mice immunized with ChAd63 and MVA expressing GFP) was included and the OD values for the negative control were less than 0.15 for all the plates tested.

### Indirect Immunofluorescence Assay

*P. falciparum* gametocytes were cultured as described previously^10^ and pelleted. For gametocyte preparations, cultures were split and half were activated in ookinete media^62^, following which samples were fixed for 10 min in 4% PFA in PBS. The non-activated and activated preparations were then pipetted onto poly-Lysine coated multiwell slides, air dried and allowed to settle for 12 h, to allow the adherence of parasites and red blood cells (RBCs) to slides. Slides were then washed three times in Tris-buffered saline (TBS) and blocked using 10% goat serum (Sigma, UK) in 1% BSA/PBS for 1 h. The slides were incubated for 1 h with day 70 mouse antisera (Pfs230-C, Pfs48/45_−NGln_, and Pfs48/45_+NGln_) or purified total IgG (2.5 mg/ml for Pfs25) diluted 1:100 in 1%BSA/PBS. After washing three times in TBS, goat anti-mouse IgG conjugated to Alexa Fluor 488 (Invitrogen, UK; 1:500 in 1% BSA/PBS) was added for 1 h, washed off in TBS and mounted with Vectashield/DAPI (Vector Labs, UK). The slides were visualized under x100 objective magnification using a fluorescence microscope (Leica DMI 3000 B, Leica Microsystems, UK). To assess antibody reactivity with native Pfs25 on ookinetes, Pfs25DR3 transgenic *P. berghei* parasites described previously^26^ expressing Pfs25 were used. Phenylhydrazine (6 μg/mL in PBS; Sigma, UK) treated female TO (Harlan, UK) mice were injected with 1 × 10^7^ Pfs25DR3 parasitized RBCs (pRBCs). Parasitized blood was harvested from the infected mouse and added 1:25 to ookinete media and left overnight at 19 °C. After 24 h, smears were fixed in 4% PFA in PBS after air-drying. Thereafter the procedure was carried out as outlined above.

### IgG Purification

Total IgG was purified from day 70 pooled mouse sera (equal volume of serum from all mice in a group was pooled irrespective of individual antibody titer) from test and control (vectors expressing GFP) groups as described previously^55^ using Protein G columns (Pierce, USA). Briefly, Protein G columns were equilibrated with binding buffer (Immunopure IgG, Pierce, USA) after which a 1:1 mixture of sera and binding buffer was allowed to flow through under gravity, washed and eluted with elution buffer (Immunopure IgG, Pierce, USA). The eluted fraction was collected in 1 M Tris-HCl (pH 9.0, Teknova, USA) and transferred for buffer exchange to Amicon centrifugal filters (Millipore, USA) using PBS. The eluent was concentrated in PBS (Invitrogen, UK) and filtered using a 0.22 μm Millipore Ultrafree sterile centrifugal unit. Total protein was quantified using a NanoDrop spectrophotometer at 280 nm and adjusted to either 2.5 mg/mL, 3.5 mg/mL or 4 mg/mL.

### Transmission-Blocking Assays

The ability of vaccine-induced antibodies to block the development of *P. falciparum* strain NF54 was assayed using gametocyte cultures as previously described^10^. In the case of cultured NF54 parasites, the percentage of mature Stage V gametocytes was adjusted to 0.15% ± 0.05% and the male-female ratio is stable (almost always 1 male : 2–3 female). These were mixed with purified IgG (diluted at 1/5.34 from adjusted total concentration of either 2.5 mg/mL, 3.5 mg/mL or 4 mg/mL giving a testing dilution of either 0.500 mg/mL, 0.655 mg/mL or 0.75 mg/mL respectively) and then fed to 4–6 day old starved female *A. stephensi* (SDA 500) via a parafilm^®^ membrane. The purified IgG was diluted in non-heat-inactivated human AB^+^ sera. Purified IgG from a group of mice vaccinated with viral vectors expressing GFP was used as negative control. Infected mosquitoes were then maintained at 26 °C and 80% relative humidity. After 7 days midguts from twenty mosquitoes were dissected, oocysts counted and the number of infected mosquitoes recorded. Percent reduction in infection intensity and prevalence were calculated relative to the control.

To determine the TBA against field isolates of *P. falciparum*, children aged between 5–11 years in Bobo-Dioulasso, Burkina Faso, were screened for the presence of asexual- and sexual-stage *P. falciparum* parasites by thick blood smears. Informed consent from parents or guardians was obtained for children positive for gametocytes (Protocol 003-2009/CE-CM, Centre Muraz institutional ethical committee). 10 mL blood was drawn in heparinized tubes to obtain gametocytes. The plasma from the gametocyte positive donor blood was replaced by AB^+^ serum from a European donor, the purified IgG were added at 0.500 mg/mL in the final mixture and immediately fed to *A. gambiae* via a parafilm^®^ membrane. Thereafter the procedure was carried out as outlined above. All SMFA and DMFA experiments were performed using non-heat-inactivated AB^+^ serum as a source of complement.

### Statistical Analysis

For the ELISA data, Wilcoxon matched-pairs signed rank test was used to determine statistical significance between pre- and post-boost (day 55 and day 70) response and Mann-Whitney test between Pfs48/45_−NGln_ and Pfs48/45_+NGln_ (day 70 responses). To determine significance of the longevity of the vaccine-induced antibody response, a Friedman test with Dunn’s correction was used to assess differences between time-points for each individual test antigen. Ability of vaccine-induced antibodies to inhibit oocyst intensity and prevalence of *P. falciparum* in transmission-blocking assays was assessed using generalized linear mixed models (GLMM) according to previously published methods^51^. A zero-inflated negative binomial error structure was used to describe oocyst intensity whilst a binomial error structure was used to determine oocyst presence or absence. Models were compared using a likelihood ratio test and confidence intervals generated using bootstrapping methodology. Significance was taken as *p* < 0.05.

## Additional Information

**How to cite this article**: Kapulu, M. C. *et al.* Comparative Assessment of Transmission-Blocking Vaccine Candidates against *Plasmodium falciparum. Sci. Rep.* **5**, 11193; doi: 10.1038/srep11193 (2015).

## Supplementary Material

Supplementary Information

## Figures and Tables

**Table 1:** **TBV candidate antigen sequences used for generation of ChAd63-MVA viral vectors.**

**Antigen**	**Accession No.**	**Full αα length**	**αα in construct**	**Features**
AgAPN1(PEST)	AGAP004809	1020	20–998	SP (1-19αα); GPI anchor(997-1020αα); No TMH; No N-glycan sites. No N-glycan sites is repeated.
Pfs230(3D7)	PF3D7_0209000	3135	443–1132	SP (1-20αα); GPI anchor(3119-3134αα); No TMH; 13 N-glycan sites.
Pfs48/45(3D7)	PF3D7_1346700	448	28–427	SP (1-27αα); GPI anchor(428-448αα); 1 TMH; 7 N-glycan sites.

Strain shown in parenthesis after rather than under antigen abbreviation; αα: amino acid; SP: signal peptide; GPI: glycosylphosphatidylinositol; TMH: Transmembrane helix.
