# Supplementary material for: Comparative Assessment of Transmission-Blocking Vaccine Candidates against Plasmodium falciparum
Source: Sci Rep. 2015 Jun 11;5:11193. doi: 10.1038/srep11193 (PMC4463016; doi:10.1038/srep11193)
Supplement: Supplementary Information [file srep11193-s1.pdf]

Comparative Assessment of Transmission-Blocking Vaccine Candidates against *Plasmodium falciparum*

M. C. Kapulu, D. F. Da, K. Miura, Y Li, A. M. Blagborough, T. S. Churcher, D. Nikolaeva, A. R. Williams, A. L. Goodman, I. Sangare[2], A. V. Turner[1], M. G. Cottingham[1], A. Nicosia[6,7,8], U. Straschil[4], T. Tsuboi[9], S. C. Gilbert[1], Carole A. Long[3], R. E. Sinden[1,4], S. J. Draper[1], A. V. S. Hill[1], A. Cohuet[2,], and S. Biswas[1]

[1]Jenner Institute, University of Oxford, Oxford, OX3 7DQ, UK.

[2]Institut de Recherche en Sciences de la Santé (IRSS) et Institut de Recherche pour le Développement (IRD) - UMR MIVEGEC, Bobo-Dioulasso, Burkina Faso.

[3]Laboratory of Malaria and Vector Research, National Institute of Allergy and Infectious. Disease / National Institutes of Health, Rockville, Maryland, USA.

[4]Division of Cell and Molecular Biology, Imperial College London, London, UK.

[5]Department of Infectious Disease Epidemiology, Imperial College London, London, UK.

[6]Okairòs AG, Rome, Italy.

[7]CEINGE, Naples, Italy.

[8]Department of Molecular Medicine and Medical Biotechnology, University of Naples Federico II, Naples, Italy.

[9]Cell-Free Science and Technology Research Centre, Ehime University, Ehime, Japan.

Supplementary Information

Design and Generation of recombinant viral vectored vaccines

All the predicted endogenous signal peptides for the antigens were replaced with the human tissue plasminogen activator (tPA) signal peptide, which has been shown to allow efficient expression of antigens, and a Kozak sequence placed upstream to maximize antigen expression in mammalian cells [1-4]. Antigen sequences were optimized to human codon usage bias and potential N-glycosylation sites were removed (except for Pfs48/45_{+NGln}). CMV long promoter with an intron A sequence (that has been shown to enhance insert expression [5]) was used to drive antigen expression in ChAd63 whilst expression was driven by the vaccinia P7.5 promoter in MVA. To generate replication-deficient, ChAd63 vaccines, the codon optimized antigen inserts were restriction cloned into pENTR™4LPTOS entry vector using Acc65I and NotI. The entry vector clone was recombined into the destination vector, ChAd63-DEST, by Gateway® cloning (Invitrogen, UK). Replication-deficient recombinant MVA vaccines were generated by cloning into an MVA.GFP shuttle vector between the thymidine kinase sites (TK_L and TK_R) by restriction cloning using Acc65I and XhoI. The resulting clone was linearized and used to generate the recombinant virus by homologous recombination into the MVA genome (parental virus, MVA-RFP). The resulting recombinant viruses were prepared and purified as previously described [6,7].

Endpoint ELISAs

Vaccine-induced antibody responses against mosquito midgut lysates were quantified by endpoint ELISAs as previously described [6]. Midgut lysates from 5-7 day old female *Anopheles gambiae* (Yaoundé and G3 strains, a kind gift from Dr George K. Christophides, Imperial

College, London) maintained under normal conditions, were prepared as previously described [8] . 5 μg/mL of the midgut lysate was used to coat Nunc-Immuno Maxisorp 96-well plates (NUNC, UK) overnight. Plates were washed (in PBS/T) and blocked (1% BSA in PBS/T) for 1 h. Test sera were diluted in PBS/T and left to incubate for 2 h after which the plates were washed in PBS/T. Secondary antibody (goat anti-mouse IgG-alkaline-phosphatase conjugated, Sigma, UK) was added at 1:5000 in PBS/T. After incubation, the plates were washed and developed by adding pNPP substrate diluted in diethanolamine buffer (Fisher Scientific, UK). The plates were read at OD_{405} until the endpoint detection set for each antigen. The cut-off for determining the endpoint titers was the average OD values for pre-immune sera + three times the standard deviation.

Supplementary Figures:

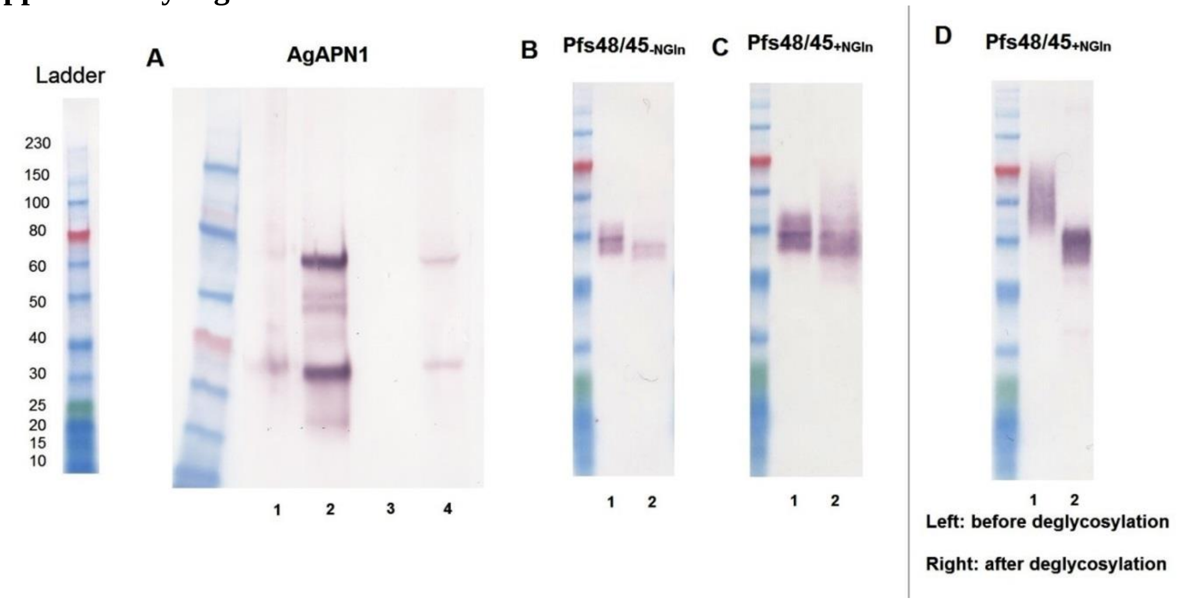

Supplementary Fig. 1. Reactivity of vaccine-induced antibodies to native antigen. *Anopheles gambiae* Yaoundé and *A. stephensi* SDA500 midgut lysate, and *Plasmodium falciparum* NF54 gametocytes purified by magnetic beads were mixed into either non-reducing or reducing SDS sample loading buffer. 5 µg/lane and 10 µg/lane of either midgut lysate or gametocyte preparation was loaded onto 8% and 12% polyacrylamide gels respectively, transferred onto nitrocellulose membranes and immunoblots obtained (after 2 min of exposure) using day 70 pooled sera from mice immunized against (**A**) AgAPN1, (**B**) Pfs48/45$_{\text{-NGln}}$ or (**C**) Pfs48/45$_{\text{+NGln}}$. Lanes: 1 – non-reducing; 2 – reducing; 3 – non-reducing; 4 – reducing buffer. (A) Lanes 1 and 2 represent *A. gambiae* Yaoundé, lanes 3 and 4 *A. stephensi* SDA500. (D) Immunoblots of supernatant of HEK293 cells transfected with pENTR™ expressing Pfs48/45$_{\text{+NGln}}$ in reducing buffer before (Lane 1) and after (Lane 2) treatment with Peptide -N-Glycosidase.

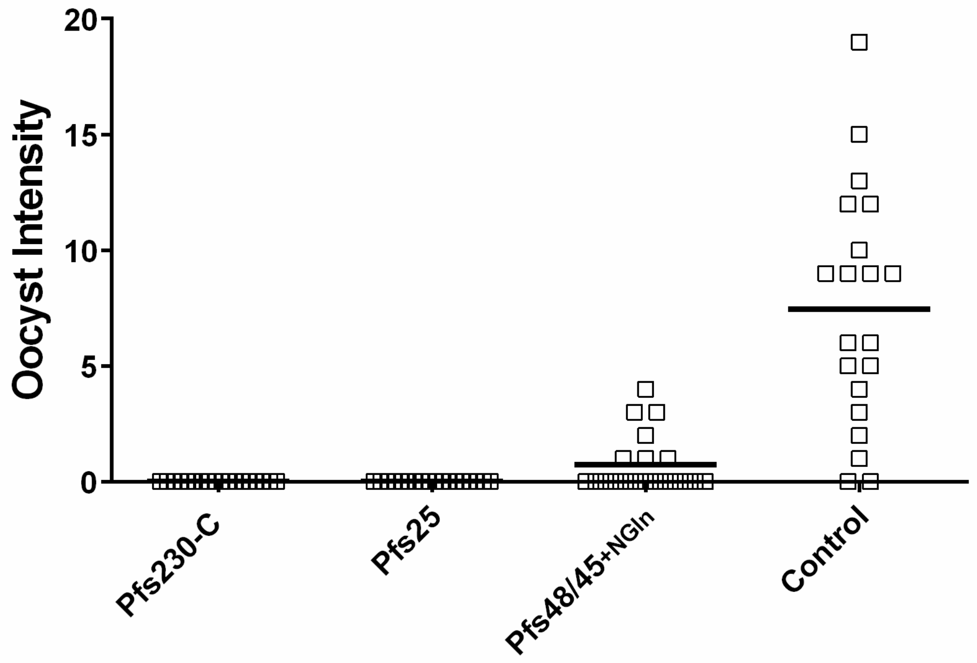

	Pfs230-C	Pfs25	Pfs48/45$_{+NGln}$	Control
N	20	20	20	20
Mean	0	0	0.75	7.45
Inhibition of Intensity (%) (lower, upper 95%CI)	100 (100, 100)	100 (100, 100)	89 (78, 95)	-
Inhibition of Prevalence (%) (lower, upper 95%CI)	100 (0.8, 1)	100 (0.8, 1)	61 (0.7, 1)	-

Supplementary Fig. 2. TBA of anti-Pfs230-C, anti-Pfs25 and anti- Pfs48/45$_{+NGln}$ IgG in SMFA. Membrane feeds were performed as described in Figure 4. Total IgG against each of the antigens (655 µg/ml) was mixed with *P. falciparum* NF54 cultured gametocytes. Midguts were dissected 7 days post-feeding. Data points represent the number of oocysts in individual mosquitoes and the lines show the arithmetic mean (from one experiment). The table shows the number of mosquitoes dissected (N) per antigen, mean number of oocysts, percent inhibition of infection intensity and prevalence calculated relative to the IgG from mice immunized with vectors expressing GFP. GLMM with a zero-inflated negative binomial error structure was used to describe oocyst intensity whilst a binomial error structure was used to determine oocyst presence

or absence. Uncertainty was generated using bootstrapping methodology. $p \leq 0.05$ was considered significant.

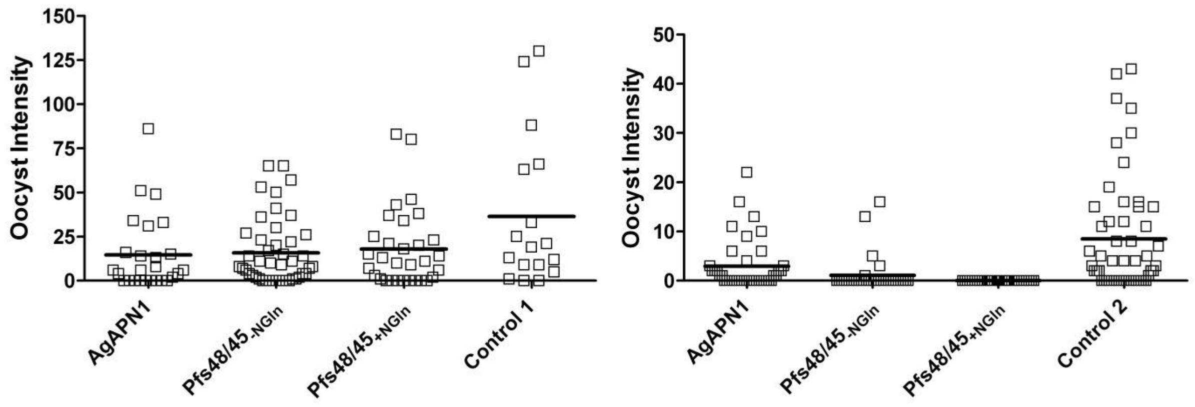

	AgAPN1	Pfs48/45$_{-NGln}$	Pfs48/45$_{+NGln}$	Control 1
N	26	45	31	17
Mean	14.6	15.8	18.1	36.4
Inhibition of Infection (%) (lower, upper 95% CI)	51 (-7, 77)	55 (10, 77)	44 (-11, 72)	-
Inhibition of Prevalence (%) (lower, upper 95% CI)	26 (-3, 71)	9 (-8, 51)	16 (-7, 61)	-

	AgAPN1	Pfs48/45$_{-NGln}$	Pfs48/45$_{+NGln}$	Control 2
N	40	35	34	53
Mean	3	1.1	0	8.5
Inhibition of Infection (%) (lower, upper 95% CI)	63 (31, 80)	82 (43, 94)	100 (100, 100)	-
Inhibition of Prevalence (%) (lower, upper 95% CI)	22 (-3, 27)	78 (24, 79)	100 (100, 100)	-

Supplementary Fig. 3. Differential inhibition of *P. falciparum* field isolates by anti-Pfs48/45$_{-NGln}$ and anti-Pfs48/45$_{+NGln}$ IgG. Membrane feeds were performed as described in Figure 5 using gametocytes from two separate donors (BF003 and BF004, control 1 and control 2 respectively). The IgG was tested at a concentration of 500 µg/mL and the gametocytes were obtained from two separate donors (separated by *y*-axis). The gametocytaemias were 31 and 15 gametocytes per 1000 leukocytes for control 1 and control 2 respectively, and the ratios of male:female gametocytes were 2.34 and 0.47 respectively. Data points represent the number of oocysts in individual mosquitoes and the lines show the arithmetic mean. The table shows the number of mosquitoes dissected (N) per antigen, mean number of oocysts, % inhibition of infection intensity and prevalence calculated relative to the IgG from vector control immunized mice. GLMM with a zero-inflated negative binomial error structure was used to describe oocyst intensity whilst a binomial error structure was used to determine oocyst presence or absence.

Uncertainty was generated using bootstrapping methodology. $p \leq 0.05$ was considered significant.

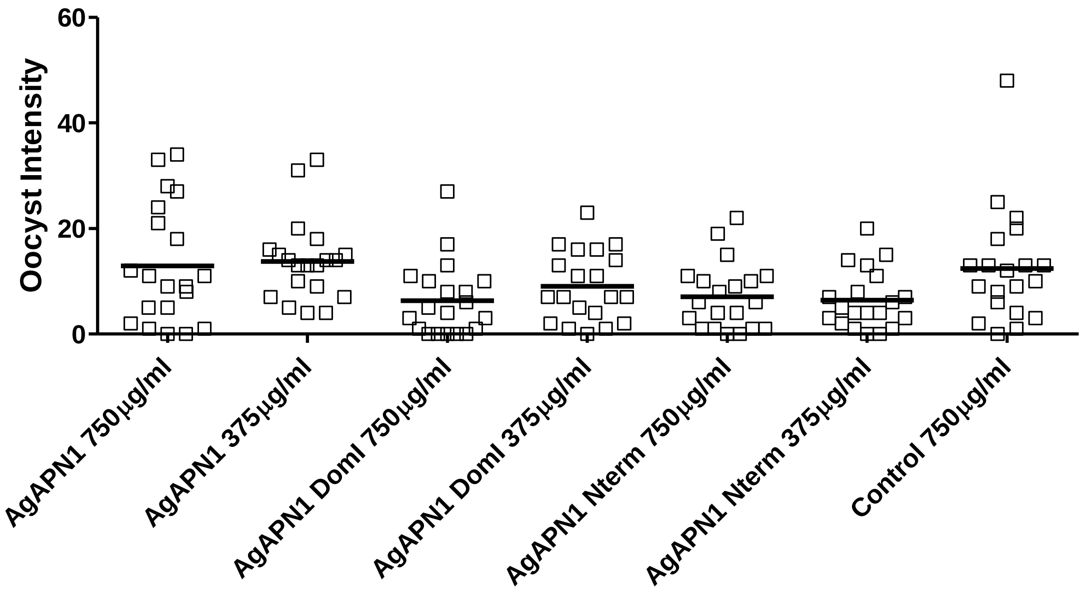

	AgAPN1 750μg/ml	AgAPN1 375μg/ml	AgAPN1 DomI 750μg/ml	AgAPN1 DomI 375μg/ml
N	20	20	20	20
Mean	13	13.8	6.4	9.1
Inhibition of Intensity (%) (lower, upper 95% CI)	-8 (-87, 38)	-7 (-63, 30)	42 (-6, 68)	28 (-20, 57)
Inhibition of Prevalence (%) (lower, upper 95% CI)	5 (-11, 20)	5 (-11, 20)	21 (0, 40)	0 (-12, 11)

	AgAPN1 Nterm 750μg/ml	AgAPN1 Nterm 375μg/ml	Control 750μg/ml
N	20	20	20
Mean	7	6	12.5
Inhibition of Intensity (%) (lower, upper 95% CI)	43 (-0.3, 67)	48 (12, 70)	-
Inhibition of Prevalence (%) (lower, upper 95% CI)	5 (-11, 20)	5 (-11, 20)	-

Supplementary Fig. 4. Effect of vaccine-induced IgG against AgAPN1 fragments to *P. falciparum* NF54 infectivity in *A. stephensi*. Membrane feeds were performed as described in Figure 4. Pooled day 70 serum (*n*=5) was used to purify total IgG against each of the AgAPN1 constructs. 750 μg/mL and 375 μg/mL of each of the total IgG was mixed with *P. falciparum* NF54 cultured gametocytes and fed to *A. stephensi* mosquitoes (*n*=20) in SMFA. Data points represent the number of oocysts in individual mosquitoes and the lines show the arithmetic mean (from one experiment). The table shows the number of mosquitoes dissected (N) per antigen, mean number of oocysts, percent inhibition of infection intensity and prevalence calculated relative to the IgG from mice immunized with vectors expressing GFP. GLMM with a zero-inflated negative binomial error structure was used to describe oocyst intensity whilst a binomial error structure was used to determine oocyst presence or absence. Uncertainty was generated using bootstrapping methodology. $p \leq 0.05$ was considered significant.

References:

1 Biswas, S. *et al.* Transgene Optimization, Immunogenicity and In Vitro Efficacy of Viral Vectored Vaccines Expressing Two Alleles of Plasmodium falciparum AMA1. *PLoS One* **6**, e20977, (2011).

2 Kozak, M. At least six nucleotides preceding the AUG initiator codon enhance translation in mammalian cells. *J Mol Biol* **196**, 947-950, (1987).

3 Golden, A. *et al.* Effect of promoters and signal sequences on the production of secreted HIV-1 gp120 protein in the baculovirus system. *Protein expression and purification* **14**, 8-12, (1998).

4 Malin, A. S. *et al.* Vaccinia expression of Mycobacterium tuberculosis-secreted proteins: tissue plasminogen activator signal sequence enhances expression and immunogenicity of M. tuberculosis Ag85. *Microbes and infection / Institut Pasteur* **2**, 1677-1685, (2000).

5 Sridhar, S. *et al.* Single-Dose Protection against Plasmodium berghei by a Simian Adenovirus Vector Using a Human Cytomegalovirus Promoter Containing Intron A. *J. Virol.* **82**, 3822-3833, (2008).

6 Draper, S. J. *et al.* Effective induction of high-titer antibodies by viral vector vaccines. *Nat Med* **14**, 819-821, (2008).

7 Cottingham, M. G. *et al.* Preventing spontaneous genetic rearrangements in the transgene cassettes of adenovirus vectors. *Biotechnol Bioeng* **109**, 719-728, (2012).

8 Dinglasan, R. R., Fields, I., Shahabuddin, M., Azad, A. F. & Sacci, J. B., Jr. Monoclonal antibody MG96 completely blocks Plasmodium yoelii development in Anopheles stephensi. *Infection and immunity* **71**, 6995-7001, (2003).
